# Effects of mutations on the molecular dynamics of oxygen escape from the dimeric hemoglobin of *Scapharca inaequivalvis*

**DOI:** 10.12688/f1000research.6127.1

**Published:** 2015-03-13

**Authors:** Kevin Trujillo, Tasso Papagiannopoulos, Kenneth W. Olsen

**Affiliations:** 1 Department of Chemistry and Biochemistry, Loyola University Chicago, Chicago, IL, 60660, USA

**Keywords:** Hemoglobin, Scapharca inaequivalvis, molecular dynamics, oxygen escape

## Abstract

Like many hemoglobins, the structure of the dimeric hemoglobin from the clam *Scapharca inaequivalvis* is a “closed bottle” since there is no direct tunnel from the oxygen binding site on the heme to the solvent.  The proximal histidine faces the dimer interface, which consists of the E and F helicies.  This is significantly different from tetrameric vertebrate hemoglobins and brings the heme groups near the subunit interface. The subunit interface is also characterized by an immobile, hydrogen-bonded network of water molecules.  Although there is data which is consistent with the histidine gate pathway for ligand escape, these aspects of the structure would seem to make that pathway less likely. Locally enhanced sampling molecular dynamics are used here to suggest alternative pathways in the wild-type and six mutant proteins. In most cases the point mutations change the selection of exit routes observed in the simulations. Exit via the histidine gate is rarely seem although oxygen molecules do occasionally cross over the interface from one subunit to the other. The results suggest that changes in flexibility and, in some cases, creation of new cavities can explain the effects of the mutations on ligand exit paths.

## Introduction

**Figure 1.**
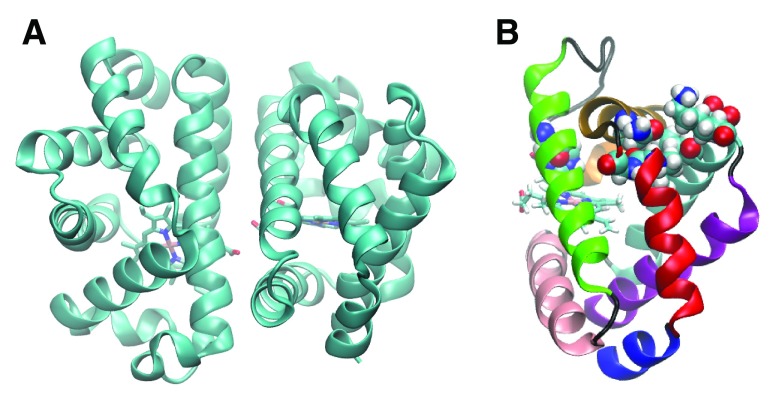
**A.** Crystal structure of HbI obtained from the protein data bank (ID: 3SDH). **B.** The helices of HbI are colored as follows: A-blue, B-red, C-ochre, D-orange, E-green, F-pink, G-cyan and H-purple. The residues shown in space-filling representation were found by implicit ligand sampling ^10^ to be on the major ligand escape. His69 is at the subunit interface on the E alpha helix, and the rest of the residues form a channel between the B and G helices; including Ile25, Asn32, Ala35, Leu36, Val121, Ser124, and Lys125. The E and F helices are involved in intra-subunit communication. The BG pathway is adjacent to the Xe4 cavity ( Figure 2B), allowing escape between these two helices.

Understanding the ligand escape pathways in the hemoglobin of *Scapharca inaequivalvis* (HbI) can provide useful insight into how structural fluctuations help facilitate ligand migration to and from the internal heme pocket. This dimeric hemoglobin has long served as a good model for studying allosteric binding due in part to its small size of 33 kDa ^1, 2^. The native clam dimeric hemoglobin structure ^3^ is shown in Figure 1a. The dimer contacts are through the E and F helices, bringing the heme groups near the subunit interface ^3, 4^. This arrangement is significantly different from tetrameric vertebrate hemoglobins ^5^. The clam dimer exhibits cooperativity with a Hill coefficient of 1.5 for oxygen binding but no response to hetrotropic allosteric ligands like bisphosphoglycerate, carbon dioxide or protons ^2^. The structural changes that occur upon ligand binding are due to intra-subunit rearrangements along the E-F helices and the displacement of interface water clusters ^3, 6^. Meta-analysis of a number of structures ^7^ produced a mechanism in which the E and F helices had strong interactions across the dimer interface allowing a small rotation but no translation within the interface during the allosteric transition. Time-dependent Raman scattering studies have established that the tertiary and quaternary changes occur simultaneously and raise questions as to how such structural changes are brought about in a concerted manner to ensure proper protein function ^8^. A combination of NMR and molecular dynamics studies on HbI ^1^ indicate that backbone flexibility contributes to both the free energy of ligand binding and the allosteric subunit rotation in the interface.

**Figure 2.**
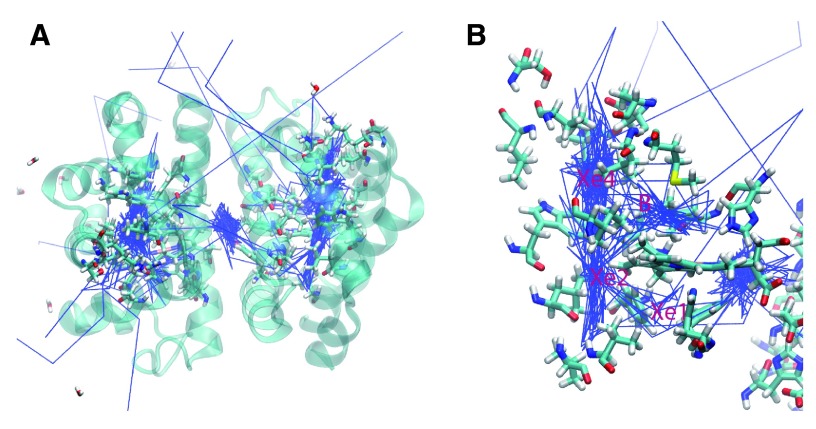
**A.** The trajectories for 14 oxygen molecules during a 10 ns simulation of the wildtype HbI are shown in blue. The main escape pathways are between the B and G and the E and F helices. One oxygen crossed over the subunit interface and another remained in the interface. **B.** The internal cavities are revealed through the presence of the dense trajectory paths. The B, Xe4, Xe2, and Xe1 cavities are shown.

HbI mutants have provided insight about how local alterations in protein structure can affect ligand binding and cooperativity. The residue Phe97 was first identified as undergoing the largest conformational change upon ligand binding by being displaced from the distal heme pocket to the subunit interface ^9^. The mutants F97V and F97L were shown to remain in the heme pocket even upon ligation due to their smaller size, while the F97Y mutant remained in the subunit interface. These experiments initially demonstrated that keeping this functionally important residue packed within the distal heme pocket leads to a significant increase in oxygen affinity ^9^. The binding rates of Phe97 mutants demonstrated that ligand induced structural changes help to facilitate ligand binding. These results could also reflect changes in structural fluctuations.

The distal histidine gate hypothesis has been proposed to explain how an oxygen molecule can enter the closed heme cavity ^10^. His69 is on the E helix and Phe97 is on the F helix, suggesting that the structural movement of Phe97 upon ligation may be initiated by swinging of the histidine gate and may also account for the mechanism of cooperativity through the E-F helices ^10^. Ligand docking sites in internal cavities were first identified in myoglobin and proposed as a means of enhancing ligand escape ^11^. Several internal cavities have been identified in HbI using xenon binding experiments to help elucidate plausible ligand migration pathways ^12^. The role of the internal cavities on ligand binding pathways has been studied in mutants of HbI. I25W and I114F were prepared in order to block ligand docking along the Xe4 and Xe2 cavities, respectively. The I25W decreased ligand escape into the solvent and had higher geminate rebinding than HBI. The Xe4 site could not be confirmed as the sole migration route because ligand escape was not completely blocked. The I114F mutant also did not completely block ligand escape. Implicit ligand sampling of the wild type structure simultaneously revealed a major pathway between the B and G helices and a minor pathway between the G and H helices that provides a direct route between bulk solvent and the internal heme pocket that depends on the structural dynamics ^10^. Although previously identified internal cavities have been dismissed from being along primary escape pathways ^10^, blocking such cavities decreased ligand escape ^13^. The residues associated with the major pathway found by implicit ligand sampling are highlighted in Figure 1b.

The position of the heme group has been demonstrated as fundamental for stable R-state interactions and is dependent on ligand-induced subunit rotation ^14^. Previous studies on HbI ^10^ have suggested the distal histidine gate as the major ligand escape pathway. Time-resolved crystallography showed that photodissociated CO rapidly bound to the distal B site and to the Xe2 and Xe4 cavities. In addition, blocking the Xe4 cavity with dichloroethane did not affect CO rebinding, suggesting that this cavity was not on a major escape route. Since the crystal lattice has a substantial effect on ligand escape, it may be that a significant conformational change or, at least, a change in conformational flexibility would be needed to allow the ligand to exit. These factors would be consistent with the histidine gate path but could be explained by other paths as well.

Previous computational studies on globins have suggested that ligand migration pathways are not conserved within this family of homologous proteins. Cohen and Schulten ^15^ used implicit ligand sampling to demonstrate different pathways in a variety of globins. Heroux, Mohan and Olsen ^16^ showed that a point mutation in a truncated hemoglobin could change the oxygen escape routes. In this study we apply locally enhanced sampling molecular dynamics ^17^ (LESMD) to visualize ligand binding in wildtype and six mutant structures of HbI. Since the bound ligand in HbI is effectively trapped in a closed bottle, the dynamics of the structure are critical to understanding ligand escape from the protein. It is useful to study the binding pathways in the clam dimer mutants compared to the native structure because mutations have been known to alter the flexibility of the protein and, therefore, may alter possible oxygen binding pathways ^18^. The crystal lattice could be limiting the flexibility of the protein in crystallographic studies, thus restricting necessary quaternary movements for ligand entry. Previous studies have argued that the crystal lattice would not affect the BG escape helices and that the protein has been found to be a very rigid structure overall, thus minimizing the effect of performing binding studies in a crystal ^10, 19^. Using molecular dynamics to study ligand escape, it was possible to visualize how the flexibility of the protein would permit a sample of oxygen molecules to explore the most energetically favorable escape pathways in the absence of crystal lattice restraints. We have been able to show how the previously identified internal cavities could facilitate ligand escape between the B and G helices, the G and H helices or other novel escape routes.

## Methods

The atomic coordinates for the native clam dimer structure and the six mutants studied were obtained from the Protein Data Bank ^20^. The crystallographic structure of the carbon monoxide bound native clam dimer at 1.7 Å was used (PDB: 3SDH ^4^). The carbon monoxide bound mutants F97L, F97V, M37V, M37F, I114F, and I25W were also obtained from the Protein Data Bank (PDB: 2AV0 ^11^, 2AUQ ^11^, 2GRH ^21^, 2R4W ^12^, 1JWN ^22^, 2R4Z ^12^). The histidines were protonated appropriately according to their environments in the protein structures. The carbon monoxide was replaced with an O _2_ ligand. The iron was parameterized as Fe (II) and bonded to the O _2_ during the equilibration of the system ^23^. For the LESMD simulations, fourteen copies of oxygen molecules were used (seven in each protein subunit). The oxygen molecules do not interact with each other and interact with the rest of the system at a scaling factor of 1/14. The protein was immersed in a water box with 0.15 M NaCl added to neutralize the charge. The cutoffs for nonbonding interactions were 12 Å. The switch distance was 10 Å, and a 1.0 1–4 scaling factor was used. The protein was equilibrated at a constant temperature of 310 K and a pressure of 1 atm using a procedure described earlier ^16^. Production simulations were performed for 10 ns at constant pressure and temperature with the 14 O _2_ ligands unbound from the iron. All LESMD calculations were performed using NAMD version 2.6 and the CHARMM27 all atom force field, while the data analysis was accomplished using Visual Molecular Dynamics software (VMD) ^23– 25^.

## Results

**Native Protein:** Analysis of the oxygen trajectories in the native structure revealed movement through internal cavities and several different escape pathways ( Figure 2). Of the 14 oxygen molecules, 5 escaped between the B and G helices, 4 between the E and F helices, 2 between the B and E helices, and 1 between the C and G helices. Two oxygen molecules went into the subunit interface through similar paths near Phe97. One of these oxygen molecules crossed completely into the other subunit in the subunit interface, while the other remained in the subunit interface for the remainder of the simulation.

The trajectories for the O _2_ ligands are shown in Figure 2. The presence of internal cavities within the protein is clearly visible, and the important residues are highlighted in Figure 2b. Three internal cavities were confirmed as migration sites within the protein. Furthermore, the oxygen molecules are largely confined within the internal cavities throughout the simulation before escaping. Prior to escape, seven oxygen molecules were in the Xe4 cavity, two were in the Xe2 cavity, two were in the Xe1 cavity, and one was in the B cavity. Before crossing into the subunit interface, one oxygen molecule was in the Xe4 cavity and one oxygen molecule was in the Xe2 cavity.

**Figure 3.**
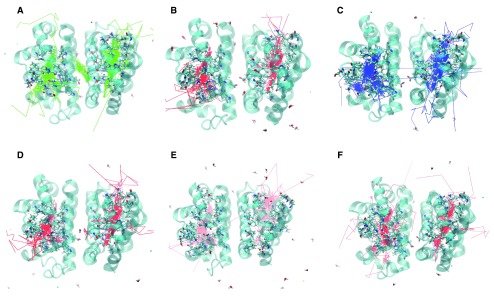
The oxygen trajectories are displayed for all 14 oxygen molecules in the six mutants studied. The trajectory for a particular oxygen molecule was recorded until it escaped the protein, “escape” being defined as more than 5 Å from the protein surface. The trajectories for oxygen molecules that failed to escape were recorded for the entire 10 ns. **A.** F97L **B.** F97V **C.** M37V **D.** M37F **E.** I114F **F.** I25W

**Figure 4.**
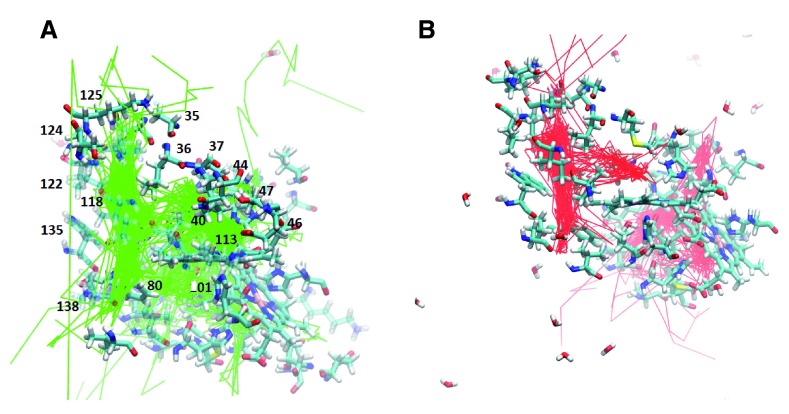
**A.** F97L trajectories highlighting the internal cavities. **B.** F07V trajectories highlighting the internal cavities. The internal cavities in each structure can be compared with the B, Xe4, Xe2, and Xe1 cavities shown in Figure 2B

**Phe97Mutants:** Due to the large structural transition of Phe97 upon ligation, there was profound effect on the oxygen escape pathways and the internal movement of the oxygen molecules within the protein cavities as a result of the structural alteration of Phe97. In the F97L simulation ( Figure 3a and Figure 4a), 3 escape pathways were identified. Six oxygen molecules escaped between the C and G helices, five between the B and G helices, and one between the A and F helices. Two oxygen molecules did not escape during the 10 ns simulation. One oxygen molecule went into the subunit interface near Leu97 before reentering the subunit from which it came. The O _2_ trajectories did not use the Xe1 cavity but more often went into the Xe2 cavity compared to the paths in the native structure. In addition, an entirely new cavity was formed adjacent to the B cavity and is bordered by the residues Tyr50, Leu40, Glu46 and Glu110. This pocket is occupied by several oxygen molecules on their exit routes but it is a dead-end and all of the ones that enter eventually return to the distal pocket before leaving the protein via another route.

 In the F97V simulation ( Figure 3b and Figure 4b), twelve oxygen molecules escaped between the B and G helices. No other escape path was evident. Two oxygen molecules failed to escape. Oxygen transport via the Xe1 cavity was attenuated, and the Val97 mutation created an additional docking site for the oxygen molecule by expanding the size of the Xe2 cavity.

**Figure 5.**
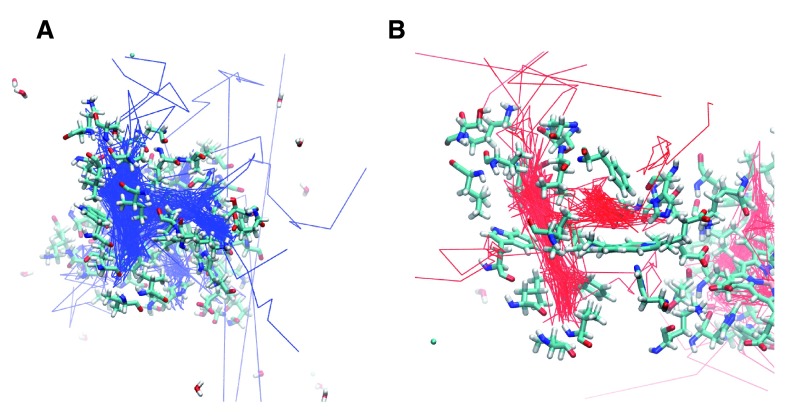
**A.** M37V trajectories showing the internal cavities used for oxygen escape. **B.** M37F trajectories reveal the internal cavities for ligand transport. The relative position of each internal cavity can be compared to the B, Xe4, Xe2, and Xe1 cavities in Figure 2B.

**Met37 Mutants:** Met37 is in the heme pocket. M37V and M37F were investigated how Met37 mutations affect oxygen escape. Several escape pathways were identified in M37V ( Figure 3c and Figure 5a). Five oxygen molecules escaped between G and H helices, three between C and G helices, two between A and F helices, and one each at the FG corner and between the C and E helices. Two oxygen molecules failed to escape, and one oxygen molecule rapidly went into the A subunit from the B subunit before crossing back over again and exiting the protein. The crossing was near Phe97. Lastly, the M37V mutation resulted in a larger B cavity, causing the oxygen molecules to spend more time in this cavity. In addition, movement along the Xe4 and Xe2 cavities was increased, while movement along the Xe1 cavity was nearly absent. There is also a novel cavity in M37V lined by the residues Gly46, Thr47, Lys113, and Ile114. One oxygen molecule was present in this cavity prior to crossing over to the other subunit and subsequently exiting between the G and H helices near the novel cavity.

Three escape pathways were identified in the M37F simulation ( Figure 3d and Figure 5b). The major escape pathway was between the B and G helices, with eleven oxygen molecules escaping through this path. One oxygen molecule escaped between the D and E helices, one between the G and H helices, and one oxygen molecule failed to escape. Two oxygen molecules entered into the subunit interface near Phe97. One of these from the B subunit ultimately escaped through that subunit, while the one from the A subunit crossed into the B subunit through the Phe97 gate. Additionally, a novel internal cavity was created near the subunit interface lined by the residues Thr72, Lys96, and His69. A few oxygen molecules lingered in this new cavity, but they were unable to cross into the subunit interface despite the cavity’s proximity to the subunit interface. Lastly, the Xe1 cavity does not appear to be an important internal migration route in the M37F mutant. Greater flexibility was also observed with respect to the residues of the B and G helices compared to the native structure, consistent with the observed structural escape pathways.

**Figure 6.**
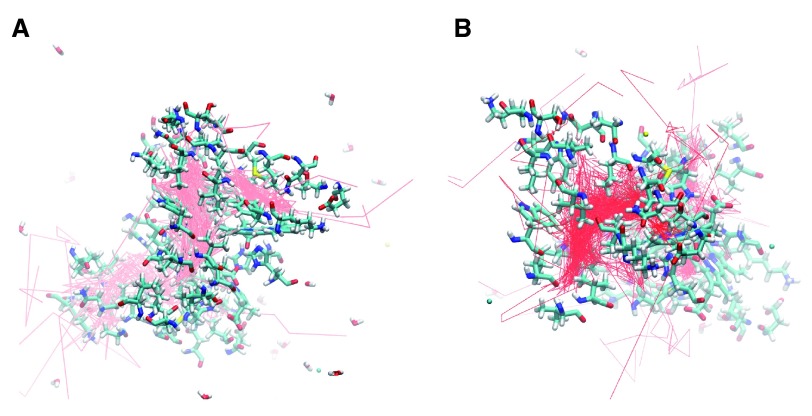
**A.** I114F trajectories showing the oxygen escape paths in pink. **B.** I25W trajectories showing the oxygen escape paths in red. The labeled residues can be compared to the internal cavity residues of the B, Xe4, Xe2, and Xe1 cavities shown in Figure 2B.

**I114F Mutant:** Ile114 is located along the path between the B cavity and the Xe4 cavity. Mutation to a larger residue such as phenylalanine has been proposed to restrict movement along this pathway ^22^. Four escape pathways were observed in the I114F simulation ( Figure 3e and Figure 6a). Six oxygen molecules escaped between the B and G helices, three between the D and G helices, two between the C and G helices, and one between the D and F helices. Additionally, the size of the Xe2 cavity was expanded to allow escape via the residues Val94, Ala7, Leu90, and Leu10. Migration into the Xe4 and Xe2 cavities was still observed, but migration though the Xe1 cavity was absent. Escape between the CG and DG helices was enhanced by the creation of an internal cavity lined by the residues Asn44, Lys113, Leu40, Tyr47, and Glu46. Lastly, only one oxygen molecule moved into the subunit interface. It migrated from the Xe4 cavity of the A subunit to rapidly escape between the B and G helices of the B subunit.

**I25W mutant:** The effect of mutation Ile25 to a larger residue such as tryptophan had previously been expected to restrict ligand docking in the Xe4 cavity ^12^. The molecular dynamics simulation of I25W ( Figure 3f and Figure 6b) showed that migration through the Xe4 cavity was restricted due to the tryptophan residue. The simulation revealed five escape pathways in total. Five oxygen molecules escaped between the C and G helices, five between the G and H helices, two between the B and E helices, and one each escaped between the C and D helices and the A and F helices. Oxygen migration became more prominent through the Xe2 and Xe1 cavities, as well as through a novel cavity lined by the residues Asn44, Lys113, Leu40, Tyr47, and Glu46.

Data of molecular dynamics trajectories. The data are represented as trajectory files (dcd) and protein structure files (psf) for each simulation. These files can be visualized using the molecular graphics program VMD, which can be obtained from http://www.ks.uiuc.edu/Research/vmd/. The files are coded by the original PDBid. WT = 3sdh, F97L = 2av0, F97V = 2auq, M37V = 2grh, M37F = 2r4w, I114F = 1jwn, and I25W = 2r4z.

## Discussion

Analysis of the native structure of the dimeric clam hemoglobin showed the presence of internal cavities ^10^. The molecular dynamics simulations reported here confirm that these internal cavities are important stops on the oxygen escape pathways. As shown in the oxygen trajectories ( Figure 2– Figure 6), the ligands spent most of the trajectory in the cavities in the native and mutant proteins. Structural alterations due to mutations cause alterations in ligand escape. New internal cavities created in several of the mutants produced novel potential escape pathways not seen in the native structure. Restricted movement within a cavity or along a tunnel due to the mutation to a larger residue also changed the potential pathways. This computational result modifies previous studies which assumed that a structural mutation to a larger residue would make the internal cavity completely unable to function ^22^. Our LESMD simulations suggest that alterations to the internal cavities as a result of the mutations alter their function rather than eliminate them.

Previous studies have used implicit ligand sampling (ILS) to identify the potential escape pathway between the B and G helices ^10^. Although this pathway has been largely ignored in favor of the histidine gate path ^10^, the LESMD simulations showed that its proximity to the Xe4 cavity allows for oxygen transport, making it a potentially important ligand escape pathway. Since ILS and LESMD use very different algorithms to predict potential pathways, the agreement of these two methods is significant. The computational results suggest pathways that are not immediately obvious from the crystal structure of HbI. Elber compared the benefits of simulations and experiments on globins ^26^ and found that simulations identify the possible escape paths, while experiments reveal the likely escape paths. The paths found in our simulations resulted from routes readily accessible between internal cavities and bulk solvent. Implicit ligand sampling studies have also shown an active role of internal cavities as important docking sites in myoglobin ^27, 28^.

**Figure 7.**
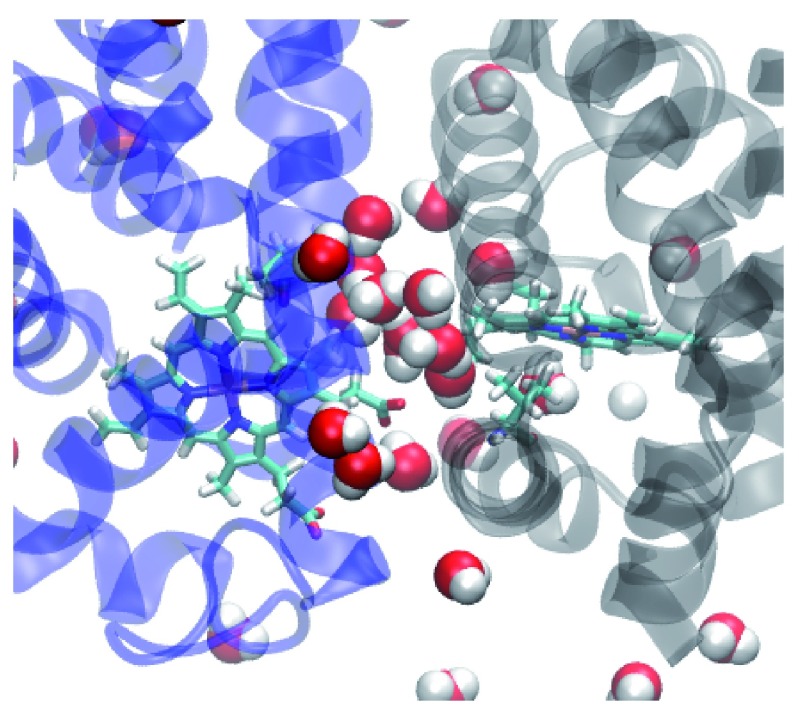
The interface of the F97V mutant showing the interfacial water molecules that are still present after 10 ns of simulation.

Crystallographic and molecular dynamics studies have revealed that a water cluster of 17 molecules at the interface rearrange in the ligated structure that could serve to enhance vibrational energy transport between subunits ^29– 32^. The stable interface interactions can be viewed as a means of transferring information and enhancing intra-subunit communication. We were able to confirm that the existence of a stable, hydrogen bonded water network within the subunit interface. This network is not destroyed by the mutations presented here. For example, at least ten water molecules stayed in the interface throughout the simulation for the F97V mutant ( Figure 7). Although the F97V had a particularly stable hydrogen-bonded water network, similar networks are found in the other proteins also. The existence of such a stable water network may explain why the oxygen molecules were not observed leaving the dimer through the interface rather than towards the bulk solvent. We did observe oxygen molecules crossing between subunit in many of our simulations. This, however, is a much rarer event in the LESMD simulations than escape between pairs of helices. The stable hydrogen bonding network of the interfacial water molecules may provide a tunnel for directing ligands across the interface. LESMD and other studies have demonstrated that globins are known to use tunnels to enhance ligand transport ^31, 33, 34^.

## Conclusion

Our molecular dynamics studies have revealed that local structural alterations can result in fluid alterations in protein transport pathways. The studies presented here suggest that multiple exit paths for oxygen exist in the wildtype and most, but not all, of the mutant HbI proteins. Thus, the interpretation of experimental changes in oxygen binding due to mutations must be done with caution. LESMD has revealed that the interfacial water clusters and hydrogen bonding network may establish a channel through which oxygen molecules may flow between subunits. LESMD has also elucidated possible escape pathways, with escape between the B and G helices being among the most common escape route. The histidine gate did not seem to be an important escape route in any of the simulations. Importantly, we have demonstrated that the internal cavities functionally enhance oxygen transport in simulations of the dimeric hemoglobin of *Scapharca inaequivalvis*.

## Data availability

F1000Research: Dataset 1. Data of molecular dynamics trajectories, 10.5256/f1000research.6127.d43528 ^35^
