# Supplementary material for: Effects of mutations on the molecular dynamics of oxygen escape from the dimeric hemoglobin of Scapharca inaequivalvis
Source: F1000Res. 2015 Mar 13;4:65. [Version 1] doi: 10.12688/f1000research.6127.1 (PMC4376171; doi:10.12688/f1000research.6127.1)
Supplement: Peer review history

# Peer review: Referee response for version 1

**DOI:** 10.5256/f1000research.6565.r7959

**Published:** 2015-03-25

**Authors:** Karin Nienhaus

**Affiliations:** 1 Karlsruhe Institute of Technology, Institute of Applied Physics, Karlsruhe, Germany

The authors present a very interesting molecular dynamics study on ligand migration within and escape from the dimeric wild-type *Scapharca inaequivalvis* HbI and various mutants. The study suggests that multiple ligand exit pathways co-exist and that the histidine gate does not serve as an important escape route in the simulations. Interestingly, very similar conclusions were drawn by Cohen *et al*. (ref. 27), who studied ligand escape from myoglobin by molecular dynamics and also found a large number of exit routes.

Experimentally, there is no direct evidence identifying the entry/exit portal for ligands into Mb or other globins. Perutz and Matthews (1966) speculated early on that rotation of the distal histidine side chain could form a channel between the heme pocket and the solvent. Later on, crystal structures of Mb with bulky ligands bound to the heme iron showed that an exit channel can be pried open by these ligands ( Ringe *et al*., 1984; Johnson *et al*., 1989). However, there are also reports arguing in favor of multiple pathways, including a high-throughput flash photolysis experiment on more than 1,500 Mb variants ( Huang and Boxer, 1994). The strongest experimental evidence of ligand entry and escape via the ‘histidine gate’ presently comes from a time-resolved x-ray crystallography study on the L29W myoglobin mutant ( Schmidt *et al*., 2005). In the mutant, ligands that became trapped in the Xe1 cavity could only escape from this cavity upon rare fluctuations of the W29 side chain that opened the path into the distal pocket. The enormously long residence time in Xe1 clearly proved that the CO in L29W had no alternative pathway available to leave the Xe1 cavity, at least on time scales relevant for the ligand binding reaction.

Flash photolysis experiments on CO-ligated wild-type *Scapharca inaequivalvis* HbI and various mutants and dioxygen equilibrium binding studies suggested that the histidine gate mechanism may hold also for this dimeric protein ( Nienhaus *et al.*, 2007). In the experiment, the internal cavities served as transient docking sites, as also seen in the simulations, but were not way stations on the exit route. Notably, in the H69L mutant and especially the H69V-I114M double mutant, the fraction of ligands that rebound geminately was significantly enhanced and bimolecular rebinding was very fast. Therefore, for me as an experimentalist, these mutants seem to be very promising candidates for additional ligand dynamics simulations because I would expect pronounced differences compared to the wt protein. I suggest to include these simulations in the present manuscript and to extend the discussion section to provide a more detailed view on the ‘histidine gate problematics’.

I have read this submission. I believe that I have an appropriate level of expertise to confirm that it is of an acceptable scientific standard, however I have significant reservations, as outlined above.

# Peer review: Referee response for version 1

**DOI:** 10.5256/f1000research.6565.r7958

**Published:** 2015-03-23

**Authors:** Andrea Mozzarelli

**Affiliations:** 1 Department of Pharmacy, University of Parma, Parma, Italy

The mansucript by Trujillo *et al.* reports on the investigation of the escape pathway of oxygen in HbI from *Scapharca inaequivalvis *carried out exploiting computational tools. Wild type and selected mutants were investigated to evidence the effects of mutations on the escaping routes as well as on the relevance of cavities where ligands can transiently bind. 

The robustness of molecular simulation results is difficult to assess. One key element for this assessment is the agreement with experimental data whenever they are available. In this case, previous kinetic studies using CO as a ligand on wild type and mutants (quoted in the manuscript), as well as oxygen binding in solution (quoted in the manuscript) and in the crystalline state ( Mozzarelli *et al.*,1996), are available. Authors should re-write the Discussion  by comparing computational and experimental data, and pointing to agreements and disagrements. This approach was followed by Bisht *et al. *(2011), Spyrakis *et al.* (2011) and Abbruzzetti *et al. *(2011) for escaping routes in AHbI from Arabidopsis thaliana.   

Further points:

Authors should indicate i) how many times the simulations with the 14 oxygen molecules were carried out, ii) which is the statistics on the different escaping routes, offering a visual indication from the most probable to the less probable escaping route.

As shown by Mozzarelli *et al. *(1996) the cooperativity is fully conserved in HbI crystal as in solution, contrary to human Hb where crystallization prevent tertiary and quatrenary transitions. This makes very interesting the computational analysis here reported because the structure used in the investigation is functionally active.

I have read this submission. I believe that I have an appropriate level of expertise to confirm that it is of an acceptable scientific standard, however I have significant reservations, as outlined above.

# Peer review: Referee response for version 1

**DOI:** 10.5256/f1000research.6565.r8021

**Published:** 2015-03-18

**Authors:** Emilia Chiancone

**Affiliations:** 1 Department of Biochemical Sciences, University of Rome "La Sapienza", Rome, Italy

This is a nicely written manuscript that provides a fairly complete ligand binding Molecular Dynamics frame to *Scapharca inaequivalvis *HbI and several functionally relevant mutants. Trajectories of biatomic ligand molecules have been carefully examined in mutated proteins and have highlighted the contributions of “native” or “mutant induced” cavities to the overall dynamics of ligand escape.

Such contributions are proposed to profoundly affect the trajectories in a non-trivial manner, by the demonstration that filling cavities with bulky amino acid side chains does not automatically result in ligand exclusion from the cavity itself.  At the same time, the simulation thus provided appears to tune down the role of a distal histidine gate that would not represent a significant escape route for the bound ligand in *Scapharca* HbI.

In this respect, the discussion section could and should be improved. It might gain a wider and more general impact by including a comparison with similar molecular dynamics studies on myoglobin in which histidine rotamers appear to exert an effect upon ligand escape dynamics.

I have read this submission. I believe that I have an appropriate level of expertise to confirm that it is of an acceptable scientific standard.
